# An attempt to understand plagiarism in Kuwait through a psychometrically sound instrument

**DOI:** 10.1007/s40979-022-00120-1

**Published:** 2022-11-14

**Authors:** Inan Deniz Erguvan

**Affiliations:** Gulf University for Science and Technology, Mubarak Al-Abdullah, Kuwait

**Keywords:** Undergraduate student, Attitude, Plagiarism, Kuwait, Questionnaire validation

## Abstract

The purpose of this study is to understand student perceptions towards plagiarism and identify some factors influencing their plagiarist behaviour to be able to develop successful strategies to promote academic integrity and prevent plagiarism. Although academic dishonesty and plagiarism have been investigated by many researchers, psychometric qualities of these data collection instruments have generally been ignored, which has resulted in a shortage of standardized and validated questionnaires in the literature. Therefore, to address this issue the researcher ran a rigorous psychometric analysis on a previously developed and psychometrically evaluated questionnaire (Attitudes Towards Plagiarism). The modified instrument was conducted on 404 students studying in a private university in Kuwait in March 2022, representing the first administration of a psychometrically established plagiarism scale in the Kuwaiti context. The statistical analysis revealed that students’ perceptions are significantly different according to the high school type they graduated from, their reasons for studying at the university, and their post-graduation career plans, whereas gender, major and year of study do not cause statistically significant differences.

## Background

Plagiarism is defined as presenting someone else's words and/or ideas as your own without giving proper credit (Ellis et al., 2018). Plagiarism, in other words, is a form of cheating and stealing (Koul et al, 2009), and it occurs when someone uses words, ideas, or work products credited to another person or identifiable source without attributing the work to the source it was obtained from for profit, credit, or gain that does not always have to be financial (Fishman, 2009). It can take several forms, such as "copy and paste" without citing the source; patch-writing; giving incorrect or incomplete citations or references; presenting or referencing a secondary source as a primary one; ghost-writing; and contract cheating (De Jager and Brown 2010; Ellery, 2008; Ellis et al. 2018; Park, 2003; Zafarghandi et al., 2012). Some studies estimate that three-quarters of university students have resorted to at least one form of plagiarism during their academic career (Brimble and Stevenson-Clarke, 2005; McCabe and Bowers, 1994).

Plagiarism is a multi-layered problem, and there is no easy explanation for why students plagiarize. Internal and external variables may both play a role in the behaviours that lead to plagiarism (Francis et al., 2015). However, the relative significance of these criteria in understanding plagiarist behaviour are still unclear (Howard et al., 2014).

Internal factors such as the desire to achieve high grades, procrastination, lack of organizational skills, fear of failing a course, lack of understanding of academic dishonesty, and a lack of regard for plagiarism as a serious offence, as well as individual factors such as gender, age, and academic background, might all play a role in the development of plagiarist behavior (McGee, 2013; Eshet et al. 2012; Jone, 2011; Kisamore and Jawahar, 2007). Newstead et al. (1996) proposed that gender, age, and academic performance influence plagiarism. According to these scholars, plagiarism is more common among males, younger students, and lower achievers. Academic achievement, age, social activities, study major, and gender are five student characteristics that are commonly linked to dishonest behavior, according to Gerdeman (2000).

Lack of established rules and mechanisms, as well as the implementation of standards addressing academic dishonesty, the honour code, and effective disciplinary proceedings, are among the possible external factors of plagiarism (Roberts and Hai-Jew, 2009; Vilchez and Thirunarayanan, 2011; Azulay et al., 2013). Similarly, educators' failure to take appropriate action when students plagiarize (McCabe, Trevino, and Butterfield, 2001) and universities' failure to provide sustainable forms of anti-plagiarism management (Sutherland-Smith, 2013) have been cited as contributing factors to the rising number of plagiarizing students. Academic dishonesty may also be compelled by social factors such as peer pressure, social and cultural attitudes, and academic dishonesty standards (Gallant and Drinan, 2006). According to Ramzan et al. (2012), social and familial pressures to get higher marks might lead students to participate in dishonest activities such as plagiarism to improve their test performance. Newstead et al., (1996) discovered six significant factors for cheating behaviour in addition to these pressures: The desire to aid a friend, fear of failure, laziness, extenuating circumstances, the chance of reaping a monetary gain, and the (mis)conception that 'everyone does it,'

Recent research has suggested that the increasing availability of electronic resources (Gullifer and Tyson 2010; Jiang, Emmerton, and McKauge 2013; Postle, 2009) exacerbates plagiarism, and there are some indications that the potential for academic cheating has indeed risen to unprecedented levels during the pandemic. Many academic dishonesty incidents are linked to the increased usage of the internet, which many scholars blame for generating "opportunities" for cheating owing to the high number of paper mills, full-text databases, and collaborative web sites (Townley and Parsell, 2004; Peytcheva-Forsyth, et al., 2018). According to Jereb et al. (2018), new technologies and the Internet have a powerful impact on plagiarism, globalisation has overcome cultural barriers and gone beyond individual and societal variables. When institutions across the world were forced to switch to online study during the pandemic, cheating incidences increased. As a result of this transition, students had more chances to conduct their coursework with the aid of the internet. Students generally have the perception that cheating in online examinations is easier than cheating in-person exams, thus, they resort to cheating more during online exams (King et al., 2009).

Students’plagiarism habits tend to be influenced by developing information-communication technologies (ICT) and the Internet, as well as other factors such as students' individual factors, academic competencies, future career plans, teaching factors, and various forms of pressure they face in their courses. A deeper look into students' views regarding plagiarism might offer us more information. Thus, the primary purpose of this study is to contribute to the development of a psychometrically sound instrument in plagiarism and to investigate students' opinions of plagiarism in a private university context in Kuwait, a country that has had little research done in this area. Understanding student perceptions towards plagiarism and identifying some factors influencing their plagiarist behaviour will help us develop successful strategies to promote academic integrity and prevent plagiarism.

In recent years, academic dishonesty and plagiarism have been investigated by many researchers. Researchers often develop questionnaires as their major data collection tool to analyze participants’ attitudes and perceptions (Mavrinac et al., 2010). However, although plagiarism is a well-documented phenomenon in the academic context with questionnaires evaluating attitudes toward plagiarism in abundance (Mavrinac et al., 2010; Bashir and Bala, 2018; Clincui, et al., 2021; Hodges, 2017; Ramdani, 2018), psychometric qualities of these data collection instruments have generally been ignored. Plagiarism research rarely includes psychometric analyses of the survey instrument (Ehrich et al., 2015) and most of the scales lack proof of solid psychometric properties (Imran and Nordin, 2013). This has resulted in a shortage of standardized and validated questionnaires in the literature. As a result, this study will also contribute to the development of a psychometrically sound instrument in measuring attitudes towards plagiarism.

The main focus of this study will be on whether students’ self-reported attitudes towards plagiarism are influenced by such variables as gender, year of study, high school, reason for studying at the university, and their career plans after graduation.

## Methodology

A questionnaire was conducted at a small private university in Kuwait with around three thousand students enrolled. The university has 6 major programs, in 2 colleges, the College of Arts and Sciences and College of Business. The data collection process was reviewed and approved by the university’s institutional review board. In March 2022, an email was sent to all faculty members, asking them to share the link to an anonymous electronic survey with their students. 404 students completed the questionnaire. Table 1 shows the demographics of the participants.

**Table 1:** Demographics of participants

Variables		n	%
Gender	Female	255	63.1
	Male	149	36.9
Major	CAS: English- Mass Comm- Comp Science	161	39.9
	CAB: Account& MIS- Econ& Finance-B Admin	243	60.1
Year	1st year	158	39.1
	2nd year	132	32.7
	3rd year and more	114	28.2
High School	Arabic	234	57.9
	English	170	42.1
Reason for studying at the university	Employment	207	51.2
	Succeed in society	136	33.7
	Learn more about topic	47	11.6
	Other	14	3.5
Plans after graduation	Further study	176	43.6
	Business/Private sector employment	114	28.2
	Government employment	52	12.9
	Other	62	15.3
Total		**404**	**100.0**

When the distribution of the participants by gender is analysed, it is seen that 63.1% of the participants are female and 36.9% are male. The distribution of the participants according to their college reveals that 39.9% of the participants are from College of Arts and Sciences, which include English, Mass Communications and Computer Science and 60.1% are from the College of Business Administration, including Accounting & MIS, Economics & Finance, and Business Administration.

The participants according to their years shows this distribution: 39.1% of the participants are within their first year at the university, 32.7% are in their 2^nd^ year and 28.2% have been studying 3 or more years. According to their high school, 57.9% of the participants are graduates of Arabic high schools and 42.1% are English. When participants were asked why they are studying at the university, 51.2% of the participants said mainly for Employment, 33.7% are studying to succeed in the society they live in, 11.6% want to learn more about their favourite topic and 3.5% are other. This other includes reasons such as ‘Their parents want them to study at the university; They had no other choice; They are here because they got accepted”, etc.

Based on their post-graduation plans, 43.6% of the participants are interested in pursuing a further study, 28.2% intend to work in the private sector employment, 12.9% of them would like to work in a government job and 15.3% of them have selected the other option, which includes responses such as, they have no plans yet, they will do nothing after graduation, they will set up their own business and work in the family business.

## Instrument

To address the lack of a robust measurement tool in measuring students’ attitudes toward plagiarism, this study administered a psychometrically evaluated instrument, Plagiarism Attitudes Questionnaire developed by Mavrinac et al. (2010) and revised by Howard et al. (2014). The permission has been obtained from Mavrinac through personal correspondence.

The original instrument had a total of 29 items and three subscales, that were positive attitudes toward plagiarism, negative attitudes toward plagiarism and subjective plagiarism norms, with acceptable reliability coefficients (each greater than 0.70, Mavrinac et. al., 2010). In a follow up study by Howard et al. (2014), the scale was modified as some items which seemed to be more relevant to students in science-based faculties (such as “a plagiarized paper does no harm to science” were reworded or removed to make the scale more applicable to a wider range of university students. The psychometric properties of this updated scale were analyzed using both traditional (confirmatory factor analysis) and item response theory models (Rasch). Howard et al (2014) concluded that according to their Rasch analyses, there were some problems with each subscale, but particularly the second subscale, which was designed to measure Justification for Plagiarism had low reliability and that the survey functioned best as two subscales. However, rather than its complete removal, they recommended modifying this subscale as justification for plagiarism serves as a significant element in understanding students’ acts of plagiarism.

The fact that Covid-19 and emergency remote teaching have brought new plagiarism issues to the forefront, the researcher of this study added eight new items (Q1, Q10, Q11, Q 18, Q19, Q20, Q21, and Q22) to the scale to broaden the range of plagiarism and to address more contemporary issues such as the use of plagiarism tools and plagiarizing in online/hybrid education. Therefore, the instrument examined in this study consists of a total of 26 items that were scored on a five-point Likert scale (1 representing strongly disagree and 5 strongly agree).

The questionnaire was responded by 289 students, in October 2021. Exploratory Factor Analysis (EFA) was employed to investigate the construct validity of the revised instrument, which suggested a two-factor solution, explaining 42.4% of the variance in the data. The only problematic item, Item 25 was loaded on both factors with a difference between factor loadings less than 0.1. This item was removed from the analysis and EFA was re-run with 25 items. The results yielded a two-factor solution with a total variance of 41.8% explained by both factors. The reliability was high in both subscales (respectively, Cronbach’s alpha = 0.93 and 0.97).

Having carried out the required validity and reliability analysis, and modified the questionnaire, the finalized version was implemented with 25 questions, in March 2022 on 404 students (See Supplementary file for the data set). Before applying the exploratory factor analysis on the new data set, the Kaiser–Meyer–Olkin (KMO) test was applied to test whether the sample size was suitable for factor analysis. As a result of the analysis, it was determined that the KMO value was 0.931. In line with this result, it was concluded that the sample adequacy was ideal for factor analysis. Values between 0.5 and 1.0 are considered acceptable as KMO values, while values below 0.5 indicate that factor analysis is not suitable for the data set in question. (Dziuban and Shirkey, 1974; Altunışık et al., 2010). In addition, when the results of the Bartlett Sphericity test were examined, the chi-square value obtained was acceptable χ2(253) = 4144.275; p < 0.05).

To reveal the factor pattern of the scale, principal component analysis was chosen as the factorization method, and varimax, one of the vertical rotation methods, was chosen as rotation. In the explanatory factor analysis performed to reveal the factor pattern of the scale, 2 items were removed from the scale (Q4, Q23) due to their low factor loading, and the remaining 23 items were collected in 2 subscales. These factors explained 46.093% of the total variance. In multifactorial designs, over 40% of the explained variance is considered sufficient (Büyüköztürk, 2007).

**Fig. 1:**
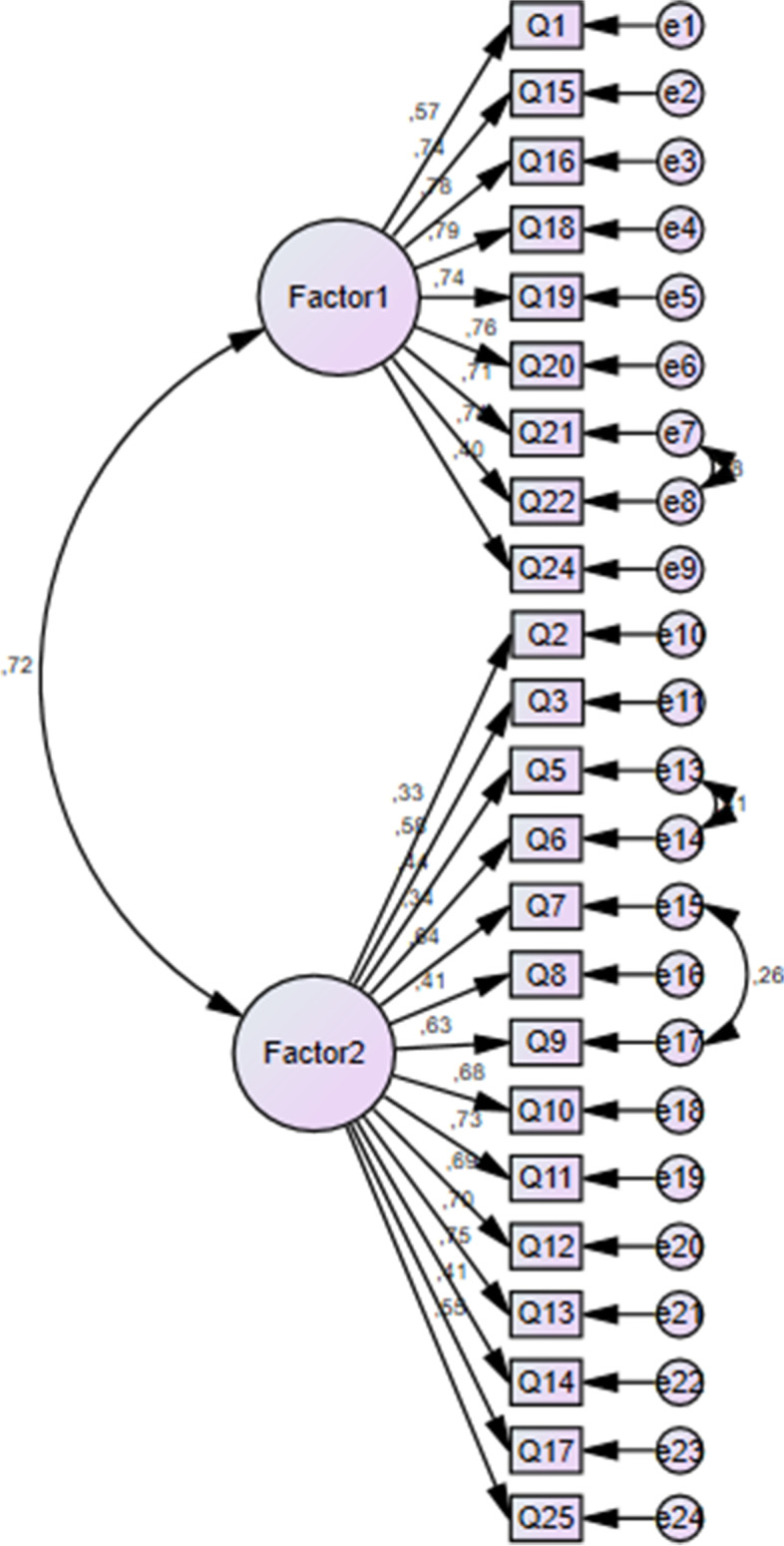
Confirmatory factor analysis

Confirmatory Factor Analysis, which could be seen in Fig. 1, was performed using the SPSS Amos program.

The correlations between the variables show that the factor loads of the items are above 0.30 and all correlation relations are significant. According to the confirmatory factor analysis, 23 items were associated with 2 subscales. Table 2 shows how the questions are distributed to two subscales.

**Table 2:** Results regarding the measurement model of the scale

Factors	Expressions	Factor loading	SE	T values	P
Factor 1 (Factors exacerbating plagiarism)	Q1.Most of my friends and classmates are plagiarising in online learning, so I feel more tempted to plagiarise	0.571	-	-	-
	Q.15 I am tempted to plagiarise if my classmate allows me to copy his or her work Q16.I am tempted to plagiarise when the punishment is light	0.740 0.783	0.116 0.114	11.183 11.561	*** ***
	Q18. When I do not have face-to-face interaction with my professors and classmates, I am tempted to plagiarize more	0.794	0.113	11.658	***
	Q19. I am tempted to plagiarize if the professor does not care about original thoughts or ideas	0.735	0.114	11.135	***
	Q20. I am tempted to plagiarize if the professor is not using plagiarism detection tools such as Turnitin	0.757	0.115	11.337	***
	Q21. When I do not have face-to-face interaction with my professors and classmates, I am tempted to plagiarize more	0.710	0.108	10.880	***
	Q22. I am tempted to plagiarize because it is easier to cheat in online education than face-to-face classes	0.735	0.118	11.122	***
	Q24. Those who say they have never plagiarised are not being honest	0.398	0.104	7.031	***
Factor 2 (Severity & Penalty of plagiarism)	Q2. First year undergraduate students are just learning the rules, so they should receive milder punishment for plagiarism	0.325	-	-	-
	Q3. Plagiarised parts of a student’s paper should be ignored if the rest of the paper is acceptable	0.580	0.296	5.878	***
	Q5. Plagiarism is as bad as cheating in an exam	0.436	0.245	5.324	***
	Q6. Plagiarism undermines & destroys independent thought and creativity	0.339	0.191	4.727	***
	Q7. Plagiarism is not a big deal as it harms no one physically	0.643	0.318	6.035	***
	Q8. Self-plagiarism should not be punished because technically you cannot steal from yourself	0.408	0.236	5.174	***
	Q9. Since plagiarism is taking other people’s words rather than tangible assets, it should not be considered a serious offence	0.629	0.274	6.002	***
	Q10. Plagiarism should not be punished under crisis conditions such as a pandemic	0.677	0.322	6.112	***
	Q11. It is OK to plagiarize when the assignment does not require me to produce anything original	0.731	0.343	6.211	***
	Q12. If you cannot write well because you do not know enough about the subject, it is OK to copy parts of a paper already written on that subject	0.685	0.338	6.128	***
	Q13. A plagiarised essay or an assignment does not harm the value of a university degree	0.703	0.329	6.161	***
	Q14. Sometimes, it is necessary to plagiarise to pass a course I am not good at	0.752	0.358	6.245	***
	Q17. Sometimes you cannot avoid using other people’s words, because there are only a few ways to describe something	0.407	0.233	5.170	***
	Q25. It is ok to use a previously written essay or assignment when the task and topic remain the same	0.554	0.272	5.797	***

^***^p < 0.05

By looking at the main theme of these factors, it was considered appropriate to call the 1^st^ subscale Factors Exacerbating Plagiarism, and the 2^nd^ subscale as Severity & Penalty of Plagiarism.

The reliability coefficients were found to be 0.892 for the first subscale (Factors Exacerbating Plagiarism), and 0.870 for the second subscale (Severity & Penalty of Plagiarism), and 0.914 for the overall scale. A Cronbach alpha value greater than 0.60 indicates that the scale is reliable, which means a high internal consistency.

## Analysis

All statistical tests were performed with Statistical Package for Social Sciences (SPSS) at the significance level of 0.05. Parametric tests (Independent–Samples t-Test and One-Way ANOVA) were selected for normal and near-normal distributions of the responses. In case of difference, Bonferroni was used to find which two groups caused the difference.

## Results

The study analysed the differences in student perceptions based on some independent variables such as gender, college, year of study, high school, reason for studying at the university and their career plans after graduation. Table 3 shows the total scale score comparison of the participants based on these independent variables.

**Table 3:** Comparison of total scale scores according to the participants’ socio-demographics

Variables		X − *X*-	SD	Test value	p
Gender	Female	62.31	13.64	0.332**	0.740
	Male	61.84	14.28		
Major	CAS: English/ Mass Comm/ Comp Sci	60.58	13.34	-1.840**	0.066
	CAB: Account& MIS/Econ & Fin/BA	63.17	14.14		
Year	1st year	61.59	14.43	0.206***	0.814
	2nd year	62.43	12.52		
	3rd year +	62.56	14.63		
School	Arabic	63.59	14.04	2.484**	0.013*
	English	60.14	13.41		
Why university?	Employment (1)	61.87	12.81	3.626***	0.013*
	Succeed in society (2)	62.26	13.91		
	Learn more about topic (3)	59.64	17.47		
	Other (4)	73.29	10.58		
Career plans	Further study (1)	59.71	13.91	3.498***	0.013*
	Business/Private sector employment (2)	64.73	12.62		
	Government employment (3)	62.79	15.01		
	Other (4)	63.73	14.08		

^*^p < 0.05, **Independent t-test, ***One-way analysis of variance

Table 3 shows the total scale scores of the participants based on their sociodemographic properties, i.e. the dependent variables of the study. According to this table and the p values of independent t-test and ANOVA, gender, major and the year of study do not yield significantly different perceptions among students. However, the type of high school they graduated from, the reason for studying in the university and career plans cause statistically significant differences in student perceptions related to plagiarism.

Table 4 displays the detailed t-test results for the high school type.

**Table 4:** Independent samples t-test for high school type

	School	n	M	SD	t	df	P
Factors exacerbating plagiarism	Arabic	234	23.3077	6.90022	1.664	402	.097
	English	170	22.1647	6.69955			
Severity & Penalty of plagiarism	Arabic	234	40.2821	8.48867	2.655	402	.008*
	English	170	37.9765	8.79346			
Total	Arabic	234	63.5897	14.03714	2.484	402	.013
	English	170	60.1412	13.40684			

^*^p < 0.05

According to the table, the high school type causes differences in student perceptions in the subscale on Severity and Penalty of Plagiarism (Factor 2). School type does not seem to cause some difference in perceptions, although not significant in Subscale 1 (Factors exacerbating plagiarism) and overall scale, but when it comes student perceptions about severity and penalty of plagiarism, those graduated from Arabic schools tend to agree more with the statements of the scale.

Table 5 shows the analysis of variance (ANOVA) results for the effects of different reasons students have for attending the university on their perceptions of plagiarism.

**Table 5:** ANOVA Result for why students are studying at the university

		SS	Df	MS	F	Sig
Factors exacerbating plagiarism	Between Groups	248.181	3	82.727	1.783	.150
	Within Groups	18,559.690	400	46.399		
	Total	18,807.871	403			
Severity & Penalty of plagiarism	Between Groups	908.392	3	302.797	4.110	.007
	Within Groups	29,472.311	400	73.681		
	Total	30,380.703	403			
Total	Between Groups	2050.581	3	683.527	3.626	.013
	Within Groups	75,407.657	400	188.519		
	Total	77,458.238	403			

Table 5 shows statistically different results within the groups for Factor 2 and total scale scores. To see which group is the cause of the difference, Bonferroni was conducted, whose results could be seen in Table 6.

**Table 6:** Bonferroni test for why students are studying at the university

Dependent Variable	(I) Reason for studying	(J) Reason for studying	Mean difference (I-J)	SE	Sig
Factors exacerbating plagiarism	Employment	Succeed in society	-.52071	.75188	1.000
		Learn more about topic	.53233	1.10062	1.000
		Other	-3.99655	1.88106	.205
	Succeed in society	Employment	.52071	.75188	1.000
		Learn more about topic	1.05304	1.15256	1.000
		Other	-3.47584	1.91191	.419
	Learn more about topic	Employment	-.53233	1.10062	1.000
		Succeed in society	-1.05304	1.15256	1.000
		Other	-4.52888	2.07399	.177
	Other	Employment	3.99655	1.88106	.205
		Succeed in society	3.47584	1.91191	.419
		Learn more about topic	4.52888	2.07399	.177
Severity & Penalty of plagiarism	Employment	Succeed in society	.12557	.94748	1.000
		Learn more about topic	1.69894	1.38695	1.000
		Other	-7.41960^*^	2.37041	.011
	Succeed in society	Employment	-.12557	.94748	1.000
		Learn more about topic	1.57337	1.45239	1.000
		Other	-7.54517^*^	2.40929	.011
	Learn more about topic	Employment	-1.69894	1.38695	1.000
		Succeed in society	-1.57337	1.45239	1.000
		Other	-9.11854^*^	2.61354	.003
	Other	Employment	7.41960^*^	2.37041	.011
		Succeed in society	7.54517^*^	2.40929	.011
		Learn more about topic	9.11854^*^	2.61354	.003
Total	Employment	Succeed in society	-.39514	1.51555	1.000
		Learn more about topic	2.23127	2.21851	1.000
		Other	-11.41615^*^	3.79162	.017
	Succeed in society	Employment	.39514	1.51555	1.000
		Learn more about topic	2.62641	2.32319	1.000
		Other	-11.02101^*^	3.85381	.027
	Learn more about topic	Employment	-2.23127	2.21851	1.000
		Succeed in society	-2.62641	2.32319	1.000
		Other	-13.64742^*^	4.18051	.007
	Other	Employment	11.41615^*^	3.79162	.017
		Succeed in society	11.02101^*^	3.85381	.027
		Learn more about topic	13.64742^*^	4.18051	.007

^*^The mean difference is significant at the 0.05 level

According to Table 6, the difference results from the group of students who has selected Other for their reason for being at the university. This group hosted such responses to the question ‘Why are you studying at the university?’ as “*My parents asked me to do it*”, “*I had no other option*”, “*I am studying because I got accepted by this university*”, “*I am here because I want to set up my own business*”. This group scored higher on the 2^nd^ subscale and the total of the scale than all the other groups, which indicates a higher level of agreement with the statement regarding plagiarism.

The next meaningful difference was observed in the sociodemographic factor of career plans of students. Student perceptions showed significant differences among students who had different post graduate career plans. Table 7 shows the ANOVA results for this variable.

**Table 7:** ANOVA Result for post-graduation career plans

		SS	df	MS	F	Sig
Factors exacerbating plagiarism	Between Groups	213.360	3	71.120	1.530	.206
	Within Groups	18,594.511	400	46.486		
	Total	18,807.871	403			
Severity & Penalty of plagiarism	Between Groups	902.486	3	300.829	4.082	.007
	Within Groups	29,478.217	400	73.696		
	Total	30,380.703	403			
Total	Between Groups	1980.434	3	660.145	3.498	.016
	Within Groups	75,477.804	400	188.695		
	Total	77,458.238	403			

One-way ANOVA was performed to compare the effect of four different career plans on students’ perceptions of plagiarism (Table 7). ANOVA revealed that there was a statistically significant difference in the mean scale score between the groups. Bonferroni was conducted to check where the difference is resulting from.

Table 8 displays that the different group is those who intend to pursue their studies with a master’s or PhD. Students with such a career plan score lower than all the other remaining groups, as their scores are lower than them. A lower score indicates a disagreement with the statements regarding plagiarism in the scale. There was no statistically significant difference between the other three groups.

**Table 8:** Bonferroni test for post-graduation career plans

Dependent Variable	(I) Career plan	(J) Career plan	Mean difference (I-J)	SE	Sig
Factors exacerbating plagiarism	Further study	Business/Private sector employment	-1.57685	.81970	.331
		Government employment	-.95236	1.07615	1.000
		Other	-1.50385	1.00693	.817
	Business/Private sector employment	Further study	1.57685	.81970	.331
		Government employment	.62449	1.14094	1.000
		Other	.07301	1.07590	1.000
	Government employment	Further study	.95236	1.07615	1.000
		Business/Private sector employment	-.62449	1.14094	1.000
		Other	-.55149	1.28209	1.000
	Other	Further study	1.50385	1.00693	.817
		Business/Private sector employment	-.07301	1.07590	1.000
		Government employment	.55149	1.28209	1.000
Severity & Penalty of plagiarism	Further study	Business/Private sector employment	-3.44099^*^	1.03207	.006
		Government employment	-2.12587	1.35497	.705
		Other	-2.51173	1.26782	.290
	Business/Private sector employment	Further study	3.44099^*^	1.03207	.006
		Government employment	1.31511	1.43655	1.000
		Other	.92926	1.35465	1.000
	Government employment	Further study	2.12587	1.35497	.705
		Business/Private sector employment	-1.31511	1.43655	1.000
		Other	-.38586	1.61427	1.000
	Other	Further study	2.51173	1.26782	.290
		Business/Private sector employment	-.92926	1.35465	1.000
		Government employment	.38586	1.61427	1.000
Total	Further study	Business/Private sector employment	-5.01784^*^	1.65147	.015
		Government employment	-3.07823	2.16815	.939
		Other	-4.01558	2.02869	.291
	Business/Private sector employment	Further study	5.01784^*^	1.65147	.015
		Government employment	1.93961	2.29869	1.000
		Other	1.00226	2.16764	1.000
	Government employment	Further study	3.07823	2.16815	.939
		Business/Private sector employment	-1.93961	2.29869	1.000
		Other	-.93734	2.58306	1.000
	Other	Further study	4.01558	2.02869	.291
		Business/Private sector employment	-1.00226	2.16764	1.000
		Government employment	.93734	2.58306	1.000

^*^ The mean difference is significant at the 0.05 level

## Discussion

The findings of our research show the effect of certain variables on students’ perceptions of the factors exacerbating plagiarism and how severely they think plagiarism should be penalized. Gender, major, or year of study seemed to have no significant impact on students’ perceptions towards plagiarism. However, the high school type based on the medium of instruction, the reason for studying at the university and students’ career plans after graduation cause statistically significant differences in their perceptions.

Gender indeed has been analysed by numerous scholars as a factor to predict plagiarist behaviour, yet there is no conclusive finding regarding the effect of gender. Although some studies find male students plagiarizing more often female students (Honig and Bedi, 2012; Guo, 2011; Martin, Rao and Sloan, 2009; Sureda-Negre, Comas-Forgas, and Oliver-Trobat, 2015), several studies reported no gender differences regarding plagiarism (Walker, 2010; Eret and Gokmenoglu, 2010; Alimorad, 2020; A. Pagaddu, 2021) or the opposite, which shows female students are more likely to commit dishonest acts (Al Suwaileh et al., 2016). The general understanding of the effect of gender on plagiarism is, academic dishonesty is context-related, and it would be too simplistic to reduce such a complex phenomenon to a simple dichotomy like gender (Bokosmaty et al., 2019).

Year of study or age and the program of study have yielded mixed results in many studies. For example, some found that younger students plagiarize more than older students (Honig and Bedi, 2012), young, male students with a poor work ethic and academic performance are more likely to plagiarize (McCabe, et al., 2001). Kincaid and Zemke (2006) point out that first and second-year male students cheat more, compared to more senior male students. However, many studies found no significant differences between plagiarism and year of study and program of study. Alimorad’s (2020) study comparing MA and PhD students did not find any significant differences between these two groups. Eret and Gokmenoglu (2010) indicate no differences between different year groups and educational programs, in their study conducted on Turkish students.

Our findings show that students who are graduates of high schools with Arabic as the medium of instruction have a significantly more lenient attitude towards plagiarism than graduates of English high schools. Students’ home or native language have appeared as a factor in many plagiarism studies previously. Bretag et al. (2018) demonstrated that a student’s language other than English makes them vulnerable to contract cheating. Also, in factors affecting why students plagiarize, students have expressed problems with using a foreign language and lack of enough academic skills are one of the reasons why they plagiarize (Eret and Goknenoglu, 2010). In a study conducted on freshman writing students, the researchers observed that students plagiarized in the course because their linguistic competencies were not sufficient to cope with the assignments (Al Darwish and Sadeeqi, 2016). Arabic high school graduates generally have difficulty producing their assignments in English in a university where the medium of instruction is English. New students in universities, students whose first language is not English or are not coming from a high school where they mastered the foreign language well, need further training for an easier transition to the research culture by understanding the practice and skills required to do research, to avoid intentional and unintentional plagiarism (Ramzan et al., 2012).

Students planning to conduct further studies scored significantly lower than their peers who had other plans such as finding a private sector or a government job or setting up their own business. Those who had academic ambitions for their post-graduation career path were more inclined to see plagiarism as a serious offence and that the students who plagiarize should be penalized more harshly. Also, students who do not have a clear reason why they are studying at the university seem to be more tolerant with plagiarism as they scored higher than other reasons for studying at the university such as finding employment and learning more about their favourite subject. Such students who have generally indicated that they are studying because it is their parents’ wish, have scored higher than the other groups which shows more tolerance towards penalizing plagiarism and not seeing plagiarism as a serious offence. Wanting to conduct advanced studies could be seen as a marker of academic motivation, and not studying at the university for a particular reason could be seen as a marker of lack of motivation. These results show us motivation and plagiarism perception are connected and highly motivated students are less likely to commit plagiarist behaviour. The results are mainly consistent with the literature. Students with higher intrinsic motivation do not tend to engage in plagiarism (Murdock and Anderman, 2006) while students motivated by extrinsic goals such as high grades and high pay rather than learning for the sake of learning tend to be more involved than students motivated by intrinsic goals (such as the desire to learn and develop their skills) (Miller and Izsak, 2017). This indicates that students’ motivation can influence their attitudes towards plagiarism. Another study shows that students with lower motivation for study spend more time on the Internet, and resort more to plagiarism as the Internet is one of the simplest solutions for studying (Jereb et al., 2018), and plagiarism.

## Conclusion and Recommendations

This study has provided data collected from a psychometrically evaluated instrument to understand the effects of gender, college of study, year in college, high school type, reason for studying in college and career plans post college and compared the data to some previously conducted studies on these variables on college students. The results indicate that gender, college, year of study do not cause significant differences ins tudent perceptions, but high school type, reason for studying in college and career plans after college do.

Our findings showed that the linguistic and research capabilities of students may be an influential factor that may determine whether a student will plagiarize or not. This highlights the importance of preparation or foundation classes of universities that conduct instruction in English and other foreign languages. Students who are not capable of producing a college-level assignment as they enrol in a university should be exposed to an intensive year of developmental language (primarily English in modern academia) program that will equip them with the required academic reading and writing skills that they will need in their major courses. Research skills should be emphasized in all courses and students who lack these essential skills should be directed to remedial classes by their professors. Thus, the primary purpose of foundation programs and freshman level courses should be to instil capabilities of writing at the college level.

Students who are intrinsically motivated to study and learn more about their courses will be less likely to resort to academic dishonesty. Those who are not, will be looking for quick shortcuts to get a good grade and graduate as quickly as possible. However, an academic environment that emphasizes honesty, ethics and values will be more nurturing even for students who are not as highly motivated to study there. If there are stringent policies against plagiarism and sanctions to follow up on those who commit academic fraud, this will be a deterrent for students.

To summarize, plagiarism is a growing threat in higher education institutions. Academic integrity violations have been common in recent years and more so during the Covid-19-mandated online or hybrid education period. Even if we return to face-to-face instruction, students are likely to stick to their tried and true, practically perfected methods of cheating. As a result, violations of academic integrity necessitate a rethinking of teaching and evaluation methodologies.

Higher education institutions must adapt to the changing plagiarism and cheating strategies that students have adopted and ensure that the faculty are aware and recognize the indicators of plagiarism and contract cheating. Students should also be given the message that their tutors are aware of plagiarism and contract cheating services. To keep up with the constant changes in technology, academic integrity processes must be current, resilient, and assessed on a regular basis.

According to McCabe (2001)), the internet is essentially a source of communication for younger students, so they have trouble understanding how to use it properly as an academic tool. High school students do not consider cutting and pasting from the internet as cheating, based on their self-reported opinions. Unfortunately, many students believe that the Internet is essentially public information and that it "does not need to be footnoted—even if it is quoted verbatim" (McCabe, 2001, p. 41). Although McCabe's research focused on high school students, when high school students go on to college, and their views tend to carry over into their college setting if their mentality does not change through guidance and proper training on academic ethics.

If we do not take immediate action, all forms of plagiarism will likely reach epidemic proportions in the very near future. We need to take a comprehensive approach that includes a focus on assessment design, a strengthened culture of integrity, and robust technical tools. We should also urge academics to perform ongoing research on ways to improve academic integrity during and post pandemic higher education instruction.

## Limitations

The author is confident this paper will add significant value to the body of existing literature; however, we cannot be sure that the working population was sufficient to capture the exact situation in other higher education institutes in Kuwait. It is also important to note that the study is limited to the experiences and assumptions of students who participated in the study and the variables presented by the researcher in the questionnaire.

## Data Availability

The dataset supporting the conclusions of this article is included within the article and the additional file.
